# Sitagliptin improved glucose assimilation in detriment of fatty-acid utilization in experimental type-II diabetes: role of GLP-1 isoforms in Glut4 receptor trafficking

**DOI:** 10.1186/s12933-017-0643-2

**Published:** 2018-01-11

**Authors:** E. Ramírez, B. Picatoste, A. González-Bris, M. Oteo, F. Cruz, A. Caro-Vadillo, J. Egido, J. Tuñón, M. A. Morcillo, Ó. Lorenzo

**Affiliations:** 1 Renal, Vascular and Diabetes Laboratory, Instituto de Investigaciones Sanitarias-Fundación Jiménez Díaz, School of Medicine, Universidad Autónoma, Av. Reyes Católicos 2, 28040 Madrid, Spain; 2 Biomedical Applications of Radioisotopes and Pharmacokinetics, Centro de Investigaciones Energéticas, Medioambientales y Tecnológicas (CIEMAT), Madrid, Spain; 3 Veterinary School, Universidad Complutense, Madrid, Spain; 4 Department of Cardiology, Hospital Fundación Jiménez Díaz, Madrid, Spain; 5 Spanish Biomedical Research Centre in Diabetes and Associated Metabolic Disorders (CIBERDEM) Network, Madrid, Spain

**Keywords:** Diabetic cardiomyopathy, Sitagliptin, GLP-1, GLP-1(9-36), Glut4, PET

## Abstract

**Background:**

The distribution of glucose and fatty-acid transporters in the heart is crucial for energy consecution and myocardial function. In this sense, the glucagon-like peptide-1 (GLP-1) enhancer, sitagliptin, improves glucose homeostasis but it could also trigger direct cardioprotective actions, including regulation of energy substrate utilization.

**Methods:**

Type-II diabetic GK (Goto-Kakizaki), sitagliptin-treated GK (10 mg/kg/day) and wistar rats (n = 10, each) underwent echocardiographic evaluation, and positron emission tomography scanning for [^18^F]-2-fluoro-2-deoxy-d-glucose (^18^FDG). Hearts and plasma were isolated for biochemical approaches. Cultured cardiomyocytes were examined for receptor distribution after incretin stimulation in high fatty acid or high glucose media.

**Results:**

Untreated GK rats exhibited hyperglycemia, hyperlipidemia, insulin resistance, and plasma GLP-1 reduction. Moreover, GK myocardium decreased ^18^FDG assimilation and diastolic dysfunction. However, sitagliptin improved hyperglycemia, insulin resistance, and GLP-1 levels, and additionally, enhanced ^18^FDG uptake and diastolic function. Sitagliptin also stimulated the sarcolemmal translocation of the glucose transporter-4 (Glut4), in detriment of the fatty acyl translocase (FAT)/CD36. In fact, Glut4 mRNA expression and sarcolemmal translocation were also increased after GLP-1 stimulation in high-fatty acid incubated cardiomyocytes. PI3K/Akt and AMPKα were involved in this response. Intriguingly, the GLP-1 degradation metabolite, GLP-1(9-36), showed similar effects.

**Conclusions:**

Besides of its anti-hyperglycemic effect, sitagliptin-enhanced GLP-1 may ameliorate diastolic dysfunction in type-II diabetes by shifting fatty acid to glucose utilization in the cardiomyocyte, and thus, improving cardiac efficiency and reducing lipolysis.

## Introduction

More than 300 million people worldwide currently suffer from type-II diabetes mellitus (T2DM) [1]. Diabetic cardiomyopathy (DCM) is defined by functional and structural changes at the myocardium, independent of any vascular or cardiac disease [2]. However, DCM and dyslipidemia-associated vasculopathies or hypertension frequently coexist in T2DM subjects, and despite access to a variety of treatments, cardiovascular diseases are the main cause of death in this population [3]. Besides of fatty acids (FA), glucose is a crucial source of energy in the heart, especially after injury. Damaged hearts shift energetic substrate toward highly efficient glucose in detriment of FA, but this metabolic flexibility is impaired under insulin resistance, leaving to FA as unique fuel provider. However, an oversupply of lipids can saturate mitochondria, producing toxic and oxidative metabolites, and cardiac dysfunction [4]. In this regard, cardiac function has been improved described in patients with heart failure after balancing lipid degradation toward glucose oxidation [5]. Also, overexpression of glucose transporter-4 (Glut4) stimulated glucose delivery, reduced FA utilization, and enhanced cardiac performance in obese/T2DM mice [6]. In consonance, deletion of FA-translocase (FAT)/CD36 receptors prevented myocyte triacylglycerol accumulation, increased glucose utilization and ameliorated cardiac dysfunction in PPARα-overexpressed mice resembling T2DM [7]. Thus, regulation of main cellular receptors for glucose and/or FA could represent a promising therapeutic target for DCM.

Sitagliptin, a dipeptidyl peptidase-4 (DPP-4) inhibitor, has demonstrated insulin dependent and independent cardio-protective actions in DCM by increasing glucagon-like peptide-1 (GLP-1), and following activation of, at least, its pancreatic and cardiac receptors (GLP-1R) [8, 9]. In pancreas, GLP-1R activation stimulated insulin secretion, whereas in the cardiovascular system, it promoted anti-fibrotic/-hypertrophic/-apoptotic responses [10, 11]. However, the effects of sitagliptin on cardiac energetic substrate uptake, in particular, on Glut4 and FAT/CD36 distribution have not been elucidated. In addition, previous data suggest that the main degradation peptide of GLP-1, GLP-1(9-36), may also interact with GLP-1R or different receptors to induce cardio-salutary actions [10, 12]. Therefore, both GLP-1 and GLP-1(9-36) could trigger direct insulin-mimetic effects on cardiomyocytes and enhance cardiac alterations associated to T2DM.

## Materials and methods

### Experimental model of T2DM

A polygenic non-obese non-hypertensive model of T2DM was used in this work. Goto-Kakizaki (GK) rats exhibit similar metabolic, hormonal and vascular disorders that the human T2DM, offering a convenient model for the study of T2DM per se, without the confounding effects of obesity or hypertension. Male GK (Taconic, Denmark) were kept on artificial 12-h light–dark cycles (7 a.m.–7 p.m.) at 25 °C. Animals had free access to chow and water. Once T2DM became well-established (at the 25th week), rats were daily treated (at 10 a.m.) with sitagliptin [Merck Sharp & Dohme (Spain), 10 mg/Kg/day] or vehicle by a gavage, as previously described [10]. Sex and aged-matched wistar rats were also examined (N = 10, each group). Body weight, diet consumption and systolic blood pressure (measured by tail-cuff method) were weekly monitored. After 20 weeks of sitagliptin-administration, plasma (collected from cava vein) and hearts were isolated (at 3–7 p.m.) under 1.5% isoflurane-O_2_ anaesthesia. Plasma glucose, lipid profile, hepatic and renal parameters were enzymatically measured in the clinical department of the Hospital. Hearts were rinsed, dried, and weighted. After atria excision, left ventricles were frozen in liquid-N_2_ for biochemical assays. These investigations adhered to the Guide for the Care and Use of Laboratory Animals (NIH Publication No. 85–23, revised 1996), and the Ethics Committee of the IIS-Fundación Jiménez Díaz Hospital granted approval for these experiments.

### Glucose homeostasis and OGTT

One day before sacrifice, blood samples were collected (from tail vein) following overnight fasting (n = 7, each group). Then, rats received the corresponding treatment and plasma was immediately obtained, after which, glucose solution (0.5 g/kg) was orally administrated. Fifteen and sixty minutes later, plasma was taken again. Glucose and insulin were measured by ELISA kits (Mercodia AB; Sweden). GLP-1 was also determined by modification of Orskov method [13]. Briefly, samples were isolated in glass tubes with DPP-4 inhibitors (Vacutainer P700, BD; USA)], mixed with 0.5 M EDTA, 10,000 UIC/ml aprotinin and absolute ethanol, and centrifuged. Supernatants were snap-frozen, lyophilized and dissolved in 0.2 M glycin-0.5% human serum albumin-500 U/ml aprotinin solution. One hundred microlitre were used for GLP-1 quantification by ELISA (Epitope Diagnostic Inc.; USA). The homeostasis model assessment (HOMA) was used to assess insulin resistance (IR) from fasting plasma glucose and plasma insulin levels as it follows:

$${\text {HOMA-IR}} = {\text{fasting plasma glucose }}\left( {\text{mM}} \right) \times {\text{fasting insulin }}\left( {{\text{mU}}/{\text{ml}}} \right)/ 2 2. 5.$$

### Small-animal PET imaging

Positron emission tomography (PET) was achieved in the animals at the end of the model in the Centro de Investigaciones Energéticas, Medioambientales y Tecnologicas (CIEMAT). All the animal experiments were approved by the Animal Ethical Committee of this institution. After 1 week of acclimatization, wistar, GK and GK-sitagliptin rats (n = 3–6) were anesthetized with 1.5–2% isoflurane in 100% oxygen. Then, [^18^F]-2-fluoro-2-deoxy-d-glucose (^18^FDG, 30–50 MBq) was injected via the tail vein. Dynamic 60-min images were acquired in list-mode using a small-animal PET scanner (Argus PET-CT, SEDECAL) and reconstructed using a 2D-OSEM (Ordered Subset Expectation Maximization) algorithm (16 subsets and two iterations), with random and scatter correction. The region of interest (ROI) templates as visualized on the late phase PET images were used for measuring ^18^FDG uptake of myocardium. Isocontour function using a threshold of 40% of maximal uptake was used to make template ROIs. ^18^FDG uptake was obtained from the ROIs between 20 and 40 min and quantified using a standardized uptake value (SUV). This was calculated according to the following equation:

$$SUV = \frac{{Activity\; concentration\; in\; VOI\; (Bq/cm^{3} )}}{{Injected \; activity\; \left( {Bq} \right) / Weight \;of\; the\; animal\; (g)}}$$

### Cardiac structure and function

Transthoracic echocardiography was performed under 1.5% isoflurane-O_2_ anaesthesia in all rats before (not shown) and after the treatment. Both M-mode and two-dimensional (2D) echocardiograms were obtained using a 12 MHz ultra-band sector transducer (En Visor-C-HD, Philips). Images were obtained from the left and right parasternal window in a supine decubitus position. The following parameters were measured and calculated from M-mode tracing: left ventricular (LV) end-diastolic diameter (LVDD), LV end-systolic diameter (LVSD), ejection fraction (EF; by Teichholz method), deceleration time and the ratio of the early (E) to late (A) ventricular filling velocities. The wall thicknesses of four segments [anterior, inter-ventricular-septum (IVS), lateral, and posterior (LVPW) walls] were evaluated on short axis 2D images. LV mass was estimated following formula [14];

$${\text{LV mass}} = 1.0 5 3 { } \times \, \left[ {\left( {{\text{LVDD}} + {\text{LVPW}} + {\text{IVS}}} \right)^{ 3} - {\text{LVDD}}^{ 3} } \right].$$

### Cultured cardiomyocytes

Mouse C2C12 myoblasts (ATCC, USA) were kindly given by Dr. Konhilas (University of Arizona, USA), and maintained in DMEM supplemented with 9% foetal calf serum, 5 mM d-glucose, 50 U/ml penicillin, and 50 μg/ml streptomycin. Before confluency, the medium was replaced to differentiation medium containing DMEM and 2% horse serum. After 4 additional days, the differentiated C2C12 cells fused into myotubes. Then, cells were switched to serum-free quiescent medium overnight before stimulation. The hyperlipidemic or hyperglycemic conditions were mimicked by 6–12 h incubation with high concentrations of a common saturated free FA (FFA) [Na^+^-palmitate (16:0), 0.12 mM], or glucose (d-glucose, 25 mM), respectively (Sigma). These concentrations were not lethal after 12 h incubation [15]. Palmitate was previously conjugated with BSA in a 3:1 molar ratio as published earlier [15]. In control cells, BSA was added as described but in the absence of palmitate. Some cells were pre-treated with GLP-1 (1 nM) or GLP-1(9-36) (0.3 nM) (Sigma) 30 min before stimulation. Wortmannin (50 nM) was added 1 h before stimulation.

### Particulate fractions and Western Blot (WB)

To quantify total cellular content of specific proteins (FABP3, PPARα, SPTLC2 and PDK4), a piece (~ 50 mg) of homogenized LV (Bullet Bender, Next Advance) was dissolved in ice-cold protein lysis buffer A (50 mM Tris–HCl pH 7.5, 1 mM EDTA, 2% SDS + 1/250 mammalian protease inhibitors). To evaluate the glucose- and FA-transporters distribution, a piece (~ 50 mg) of homogenized LV was suspended in ice-cold lysis buffer B (2 mM EDTA, 2 mM phenyl-methyl-sulfonyl fluoride, and 1 µM pepstatin A in PBS). Cytosolic proteins were collected in supernatant after cold centrifugation at 50,000*g* for 30 min. The pellet, with sarcolemmal proteins was suspended in ice-cold lysis buffer B. In vitro, 50 μg of cell extracts from cultured cardiomyocytes were homogenized in ice-cold buffer C containing 1 mM EDTA, 250 mM sucrose, and 10 mM Tris, pH 7.5. The homogenates were cold-centrifuged (5 min at 760*g*), and supernatants were isolated and centrifuged (1 h at 31,000*g*) to pellet the sarcolemma enriched-fraction. Then, the second supernatant was subjected to centrifugation (1 h at 190,000*g*) to pellet the endosome enriched-fraction.

Equal amounts of all protein extracts were loaded and separated on polyacrylamide gels, transferred to membranes (iBlot, Thermo Fisher), and probed with specific primary antibodies anti-FABP3, -GPAT1, -SPTLC2 (Aviva System Biology), -PDK4, -PPARα (Sigma Aldrich), -Glut4, -Glut1, -FAT/CD36, -phospho-AKT(Ser473), -phospho-AMPKα1(Ser496) or -phospho-IRS1(Ser307) (Thermo Fisher). Anti-GAPDH or anti-pan-cadherin (Sigma) were used as loading control for cytosol or sarcolemma, respectively. Then, secondary antibodies (GE Healthcare) were used for chemo-luminescence development. A representative gel of the rats or at least three independent experiments with the semi-quantification scores (n-fold) are shown.

### Quantitative-PCR (QPCR)

Total RNA was extracted from homogenized ventricle (~ 50 mg) or cultured cardiomyocytes by dissolving in Trizol reagent (Thermo Fisher). Equal amounts of RNA were reverse-transcripted to obtain the cDNA for multiplex QPCR, as previously described [15]. The gene expression assays were Glut4 (Mn00436615_m1), FAT/CD36 (Rn00580728_m1), acyl-CoA dehydrogenase long chain (ACADl) (Rn00562121_m1), acyl-CoA dehydrogenase medium chain (ACADm) (Rn00566390_m1) and CPT1b (Rn00682395_m1) Fam-fluorophores. The housekeeping gene was eukaryotic ribosomic 18s Vic-fluorophore (4310893E). Amplification conditions were: 2′ at 50 °C, 10′′ at 95 °C and 40 cycles of 15′′ at 95 °C and 1′ at 60 °C. All samples were prepared in triplicate to obtain their threshold cycle (Ct). If deviation for each triplicate were higher than 0.3 cycles, Ct was not considered. The relative expression for each gene was achieved following the model R = 2^−ΔΔCt^. We show the average quantification (-fold gene vs. 18s) of two QPCRs of all rats or three independent cultured cardiomyocytes assays.

### Statistical analysis

Data are expressed as mean ± standard deviation. Multiple comparisons were performed by non-parametric Kruskal–Wallis test followed by a Mann–Whitney test. Statistical significance was defined from p < 0.05.

## Results

### Sitagliptin attenuated hyperglycemia and insulin resistance in GK rats

**Fig. 1:**
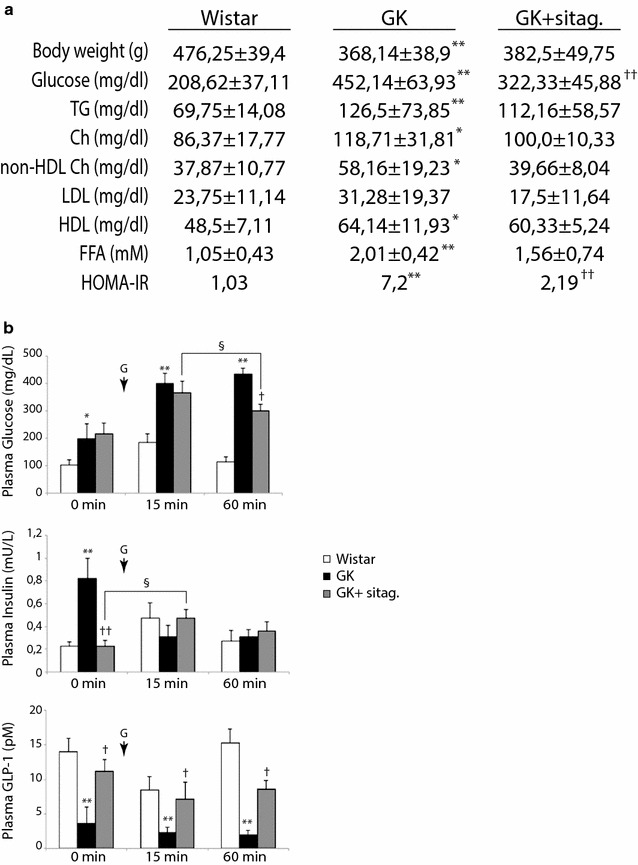
Sitagliptin reduced hyperglycemia and glucose intolerance in GK rats. **a** Body weight and plasma parameters were evaluated in the experimental model. TG, triglycerides; Ch, cholesterol, LDL and HDL, low- and high-density lipoproteins, respectively, and FFA, free FA. N = 10, each group. **b** Plasma glucose, insulin and GLP-1 were measured in fasting rats before (0 min) and after (15–60 min) an oral glucose loading (G). *p < 0.05 and **p < 0.01 vs. wistar. ^†^p < 0.05 and ^††^p < 0.01 vs. GK rats. ^§^p < 0.05 vs. 15 min glucose-overload or fasting state. N = 7, each group

Compared to aged-matched wistar, GK rats exhibited a weight loss and elevation of circulating levels of glucose and lipids [triglycerides (TG), cholesterol (Ch), non-HDL Ch, high-density lipoproteins (HDL), and FFA] (Fig. 1a). Interestingly, 20 weeks of sitagliptin administration (10 mg/Kg/day) significantly attenuated hyperglycemia (Fig. 1a), though only tended to improve hyperlipidemia. A glucose tolerance test was also achieved at the end of the model. In a fasting state, GK exhibited higher levels of plasma glucose and insulin, and reduced released of GLP-1 (0 min; Fig. 1b), suggesting insulin resistance. After 15 min of oral glucose loading, GK rats did not respond by adequately increasing GLP-1 and insulin (Fig. 1b, middle and bottom), leading to uncontrolled glycemia (Fig. 1b, top). However, sitagliptin-treated GK reacted after 15 min-glucose overload by stimulating GLP-1 and insulin, and thus, decreasing glycaemia by 60 min. In addition, the HOMA-IR index, a reliable measure of insulin resistance in human and rat [16], was elevated in GK rats and returned after sitagliptin (Fig. 1a). Of note, plasma ions (Na^+^, Cl^−^ and K^+^), markers of severe renal (urea, blood urea nitrogen, creatinine and albumin) and liver (aspartyl and alanine aminotransferases) injury, and systolic blood pressure remained within the normal ranges in all groups (not shown).

### Sitagliptin improved glucose uptake and diastolic dysfunction in the GK myocardium

**Fig. 2:**
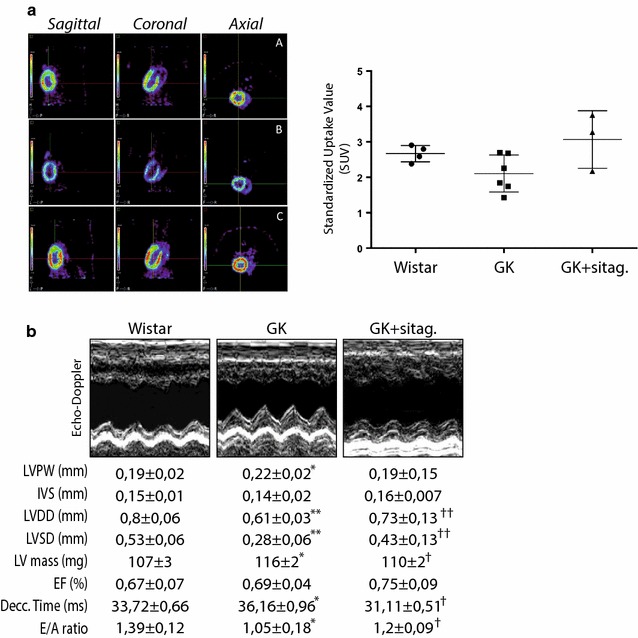
Sitagliptin improved myocardial glucose uptake and mitigated myocardial dysfunction in GK rats. **a** Left, cardiac PET images (sagittal, coronal and axial slices) of [^18^F]-FDG metabolism in (A) wistar, (B) GK and (C) GK-treated rats. Color scale bars represent the standardized uptake value (SUV) range in the myocardia. Right, quantification of SUV in the animal model. **b** The left-ventricular posterior wall (LVPW) and inter-ventricular septum (IVS) thicknesses, left-ventricular diastolic and systolic diameters (LVDD and LVSD, respectively), the ejection fraction (EF), deceleration time, and the E/A ratio were measured in the rats. Representative Echo-Doppler images for each group are also shown (top). *p < 0.05 and **p < 0.01 vs. wistar. ^†^p < 0.05 and ^††^p < 0.01 vs. GK rats. N = 8, each group

Since sitagliptin attenuated hyperglycemia and insulin resistance, we examined whether this action may affect glucose uptake by the myocardium. In fact, myocardial ^18^FDG uptake was decreased by 27% in GK rats compared to control rats although the difference was not statistically significant (SUV: 2.105 ± 0.521 vs. 2.667 ± 0.232, p = 0.06) (Fig. 2a). However, sitagliptin-treated rats exhibited a dramatic increase of the glucose assimilation in the myocardium (3.068 ± 0.811).

Next, a defect in glucose uptake in T2DM hearts could originate cardiac dysfunction [16]. Thus, by 2D-Echo-Doppler we evaluated cardiac dimensions and function in GK rats (Fig. 2b). GK exhibited a significant increase of the LV posterior wall (LVPW) thickness, and a reduction of LV diastolic and systolic diameters (LVDD and LVSD, respectively). Accordingly, LV mass was elevated in GK myocardia. As expected [10], GK rats preserved the ejection fraction, but they prolonged the deceleration time and diminished the E/A ratio, suggesting diastolic dysfunction (Fig. 2b). Interestingly, sitagliptin attenuated these hypertrophic parameters, and lessen diastolic dysfunction.

### Sitagliptin stimulated Glut4 translocation in detriment of FAT/CD36 in GK hearts

**Fig. 3:**
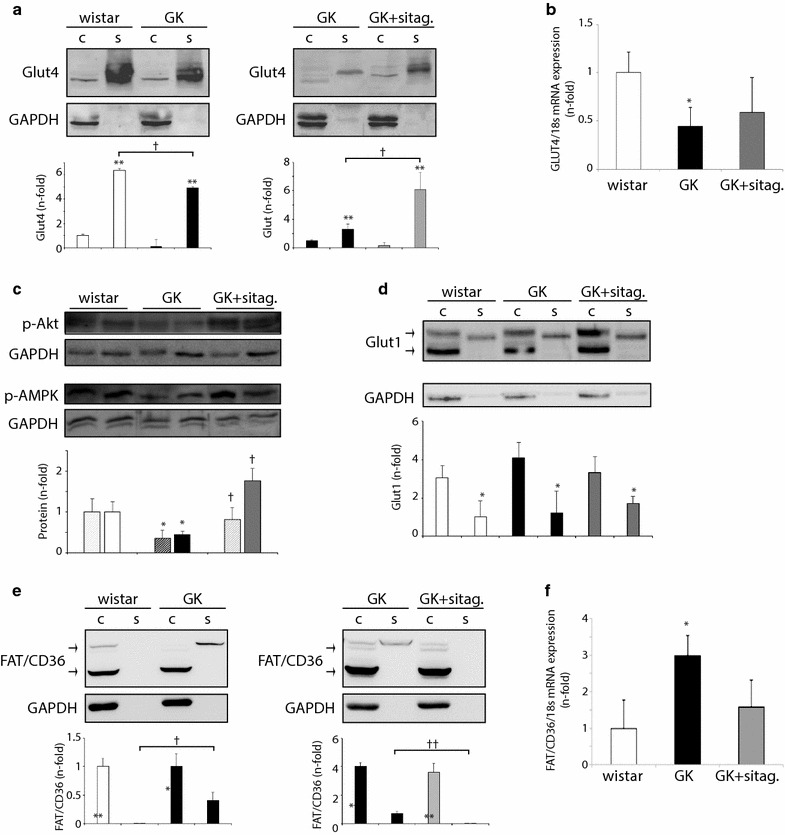
Sitagliptin enhanced cardiac Glut4 in detriment of FAT/CD36 in GK rats. **a** Glut4, **d** Glut1 and **e** FAT/CD36 protein levels in cytosolic (c) and sarcolemmal (s) fractions from wistar, GK and GK-sitagliptin myocardia. **b** Glut4 and **f** FAT/CD36 mRNA expression in the model. **c** phospho-Akt^473^ (dotted bars) and phospho-AMPKα^496^ (plain bars) protein expression in these hearts. *p < 0.05 and **p < 0.01 vs. cytosolic isoform or wistar rats. ^†^p < 0.05 vs. GK rats or sarcolemmal isoform in wistar. N = 8, each group

The stimulation of myocardial glucose uptake induced by sitagliptin may be dependent on glucose transporter trafficking. After stimulation, both Glut4 and Glut1 receptors are synthetized in the cytosol and translocated from endosome stores to the sarcolemma for glucose assimilation [17]. In wistar rats, we detected most of Glut4 in sarcolemmal fractions (Fig. 3a, left). However, GK myocardia showed a reduction in sarcolemmal, but not cytosolic, Glut4 isoform (Fig. 3a, left), and also, downregulation of Glut4 transcripts (Fig. 3b). Sitagliptin did not change cytosolic or mRNA Glut4, but it did restore sarcolemmal levels of Glut4 (Fig. 3a, right) and phospho-Akt^473^ and phospho-AMPK^496^ levels (Fig. 3c), which may explain the increased glucose uptake observed by PET after treatment. Phospho-Insulin receptor substrate-1 (IRS-1^307^) was, however, unchanged in the myocardia (not shown). In addition, Glut1 was also localized in the cytosol and in a lesser extent in the sarcolemma of wistar, but these isoforms were not significantly modified either in GK or GK-treated rats (Fig. 3d).

Since glucose and FA metabolism could be tightly coupled and inversely regulated [18], we next analysed the levels of the most relevant FA transporter in the myocardium. FAT/CD36 also synthetizes in the cytosol and moves away from endosome vesicles to the sarcolemma for FA uptake [17]. In this model, we detected FAT/CD36 mainly in cytosolic fractions of wistar rats, but it was partially shifted to sarcolemmal location in GK (Fig. 3e, left). In parallel, FAT/CD36 mRNA (Fig. 3f), but not its cytosolic isoform (Fig. 3e, left), was increased. Interestingly, sitagliptin attenuated sarcolemmal FAT/CD36 translocation (Fig. 3e, right), suggesting a potential counterbalance with stimulated Glut4 transporters.

### GLP-1 isoforms improved Glut4 translocation in HF-stimulated cardiomyocytes

**Fig. 4:**
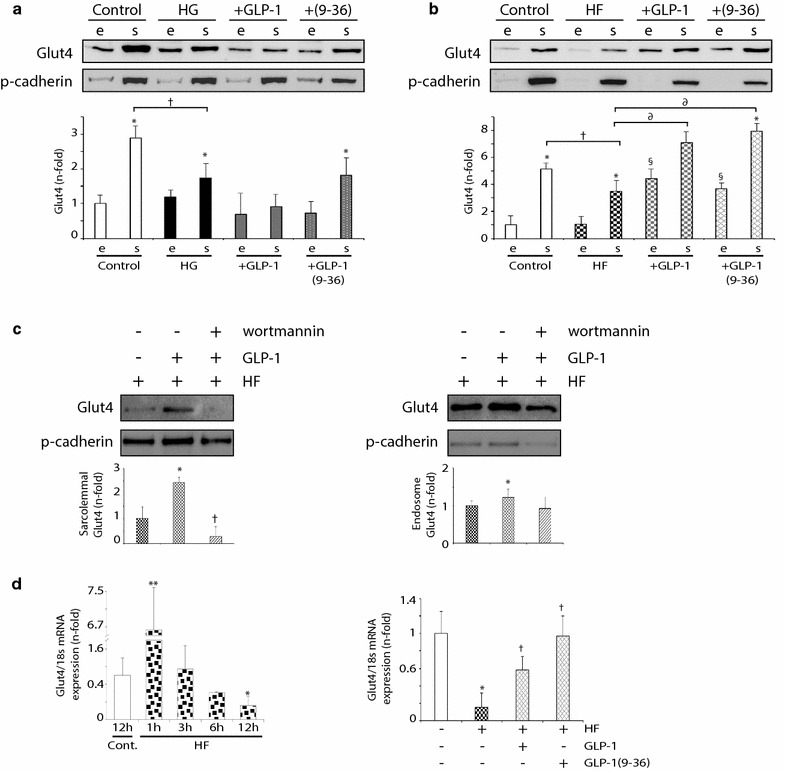
GLP-1 and GLP-1(9-36) increased endosomal and sarcolemmal Glut4 in hyperlipidemic cardiomyocytes. Glut4 expression in endosome- (e) and sarcolemmal (s)-enriched fractions of **a** HG- and **b** HF-stimulated C2C12. Some cells were pre-treated with GLP-1 or GLP-1(9-36). *p < 0.05 vs. endosome. ^†^p < 0.05 and ^∂^p < 0.05 vs. sarcolemmal. ^§^p < 0.05 vs. endosome from HF-incubated cells. **c** Sarcolemmal (left) and endosome (right) Glut4 expression was detected in Wortmannin pre-incubated GLP-1-stimulated cardiomyocytes, under HF medium. *p < 0.05 vs. HF, ^†^p < 0.05 vs. HF + GLP-1. **d** Left, Glut4 mRNA expression after HF incubation (1–12 h). Right, Glut4 mRNA expression after 12 h HF stimulation and/or GLP-1 or GLP-1(9-36). *p < 0.05 and **p < 0.01 vs. control. ^†^p < 0.05 vs. HF-incubated cells

The beneficial effects of sitagliptin on cardiac glucose assimilation may be derived from incretin-associated stimulation of insulin secretion and sensitivity, and/or from incretin direct actions on cardiac cells [10, 11]. Thus, we stimulated C2C12 cardiomyocytes with GLP-1 under high concentrations of glucose (HG) or FA (HF), and quantified Glut4 distribution in endosomes and sarcolemma. Like in hearts, Glut4 was mainly located in sarcolemma fraction. Intriguingly, after 12 h of HG (Fig. 4a) or HF (Fig. 4b), Glut4 reduced its sarcolemmal expression with no significant changes in endosome location. However, GLP-1 stimulation enriched both sarcolemmal and endosome isoforms under HF environment (Fig. 4b). Furthermore, similar effects were shown by pre-treatment with the main GLP-1 metabolite of DPP-4 activity, GLP-1(9-36) (Fig. 4b). In order to know whether PI3K pathway could be involved in these responses, we also pre-treated some cardiomyocytes with Wortmannin, a specific PI3K inhibitor. Interestingly, Wortmannin attenuated sarcolemmal Glut4 expression induced by GLP-1 (Fig. 4c, left) and GLP-1(9-36) (not shown), under HF medium. However, its endosome expression did not decrease significantly (Fig. 4c, right).

Consequently, in order to explain the endosomal increase of Glut4 after GLP-1 and GLP-1(9-36) incretins in HF milieu, we quantified Glut4 mRNA expression by QPCR in the cardiomyocytes. Glut4 transcripts were initially triggered by HF, but progressively decreased up to 12 h (Fig. 4d, left). Importantly, both GLP-1 and GLP-1(9-36) recovered Glut4 mRNA levels after 12 h of HF incubation (Fig. 4d, right).

### Sitagliptin reduced FA utilization in GK myocardia

**Fig. 5:**
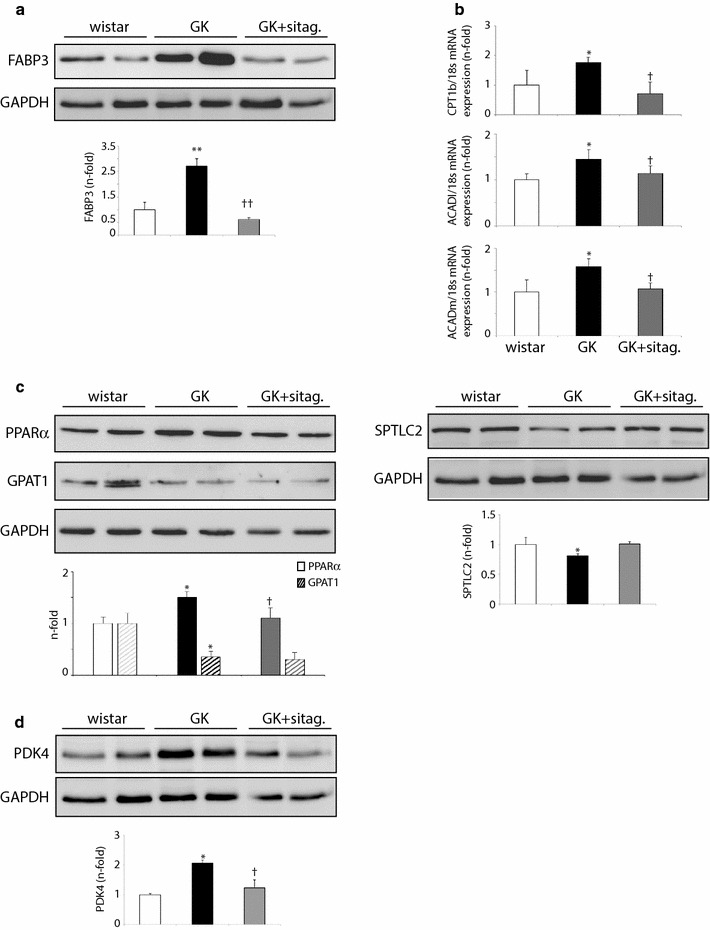
Sitagliptin reduced FA-delivery and FAO mediators in GK rats. Protein expression of **a** FABP3, **c** PPARα, GPAT1, SPTLC2 and **d** PDK4 in wistar, GK and GK-sitagliptin treated myocardia. **b** mRNA expression of CPT1b mitochondrial transporter and ACADl and ACADm FAO enzymes. *p < 0.05 and **p < 0.01 vs. wistar. ^†^p < 0.05 vs. GK. N = 8, each group

The enhanced glucose uptake and the increased balance of sarcolemmal Glut4 over FAT/CD36 in sitagliptin-treated rats may promote a decrease in FA utilization [17]. Due to the short half-life of radiolabelled FA tracers, we could only quantify the FA assimilation by indirect measurement of downstream mediators for FA-transport, -oxidation and -storage. Interestingly, the GK-induced over-expression of a key FA cytosolic carrier named FA-binding protein-3 (FABP3), was significantly restored after sitagliptin (Fig. 5a). Also, the GK-associated increase of carnitine palmitoyl transferase-1b (CPT1b) mRNA expression, a mitochondrion transporter for FA, was ameliorated by sitagliptin (Fig. 5b). Downstream, two central enzymes of FA oxidation (FAO), ACADl and ACADm, showed a similar response (Fig. 5b). Furthermore, a common transcription factor inducer of FAT/CD36, CPT1b, ACADl and ACADm expression, peroxisome proliferator-activated receptor-α (PPARα) [19], was also reduced after sitagliptin administration (Fig. 5c, left). However, lipid re-synthesis and ceramide formation may not be triggered in GK myocardia, and thus, not affected by sitagliptin. In this sense, glycerol-3-phosphate acyltransferase-1 (GPAT-1) and serine palmitoyltransferase-C2 (STPLC2), the first rate-limiting enzymes of triacylglycerol and ceramide biosynthesis, respectively, were not stimulated in GK rats and were unchanged after treatment (Fig. 5c).

Finally, the expression of a crucial interconnecting enzyme between FA and glucose metabolic pathways was studied. Pyruvate dehydrogenase kinase-4 (PDK4), a PPARα-dependent inhibitory enzyme of the glycolytic pyruvate dehydrogenase complex [19], was triggered in GK myocardia but restored after sitagliptin administration (Fig. 5d). This data enforces the potential cardiac anti-lipolysis action of sitagliptin by restoring the balance toward glucose utilization in GK rats.

## Discussion

According to the Framingham Heart Study, the risk of heart failure in diabetes is increased 2.4-fold in men and fivefold in women compared to non-diabetic subjects [20]. In this study, we observed that non-hypertensive non-obese T2DM (GK) rats exhibited hyperglycemia and hyperlipidemia, inefficient production of insulin and GLP-1, and also cardiac dysfunction. Importantly, this systemic insulin resistance may be mirrored at the myocardium. By PET, GK decreased cardiac glucose assimilation which was paralleled to a reduction in sarcolemmal content of Glut4 and Akt/AMPKα activation, while FAT/CD36 was elevated. Similar data were observed in T1DM [21] and obese-T2DM [22–27] animals. These data suggest that heart may suffer from insulin resistance in obesity and T2DM, and FA could be preferred as energetic substrate in these conditions. In this regard, metabolic mediators of FA utilization may be upregulated. We observed that cytosolic and mitochondrial FA-transporters (FABP3 and CPT1b), and FAO enzymes (ACADl and ACADm) were over-expressed in GK myocardia. Moreover, a related transcription factor such as PPARα, and a glycolytic inhibitor such as PDK4, were also stimulated. Thus, under glucose deficit and insulin resistance, most FA could be used for FAO and energy consecution in the heart. In this sense, we did not observe stimulation of GPAT-1 and SPTLC2 as rate-limiting enzymes of DAG and ceramide formation, respectively. However, excessive FAO could lead to ROS accumulation and mitochondrial uncoupling, becoming a maladaptive response that rise lipotoxicity and reduce energy efficiency [18]. In fact, our GK rats revealed cardiac hypertrophy and diastolic dysfunction.

Unfortunately, there is not an efficient and specific treatment for DCM. Intensive glycemic goals have failed to prevent cardiac complications in long-term diabetic patients or have even increased cardiovascular mortality [28]. New therapeutic strategies capable of preserving heart function while contributing to the overall care of diabetes may be required. In this line, a correction of the metabolic imbalance has led to positive outcomes. A reduction of cardiac FAO and/or stimulation of glucose oxidation improved heart failure, ischaemic injury, and DCM in mice and patients [29, 30]. In this work, sitagliptin, a DPP-4 inhibitor that prevents GLP-1 degradation into GLP-1(9-36) and other metabolites, reduced hyperglycemia and insulin resistance, and increased GLP-1 levels in fasting and non-fasting states, as previously described [8, 31]. More importantly, sitagliptin stimulated cardiac glucose assimilation and enriched Glut4 (but not Glut1) at sarcolemma locations, in detriment of FAT/CD36. Similarly, Vyas et al. described that exenatide, an agonist of GLP-1R, increased total Glut4 whereas Glut1 was unchanged [32]. Although Glut1 could compensate Glut4 lessening at least in hypertensive and hypertrophied hearts [33, 34], Glut1 is constitutively present on plasma membranes of multiple tissues including myocardium [35]. In consequence, FA utilization might be also reduced after sitagliptin administration. Indeed, the expression of FABP3, CPT1b, and ACADl and ACADm, was diminished in GK-treated hearts. Also, PPARα and PDK4, were lessened. Thus, sitagliptin-stabilized GLP-1 may improve glucose assimilation, and co-ordinately, decline excessive FA utilization and subsequent potential mitochondrial saturation and ineffectiveness. By balancing metabolic and oxidative state, diabetic hearts could enhance their structure and performance [36]. In fact, GK-treated rats exhibited an improvement of cardiac hypertrophy and diastolic dysfunction. However, despite evidence from clinical studies have demonstrated that sitagliptin could exert cardioprotection after specific heart injury (i.e., ischemia) [37], this inhibitor could not reduced LV diastolic dysfunction and lipid profile in T2DM subjects [38]. Likely, both the presence of different DPP-4-targets than GLP-1 and the absence of GLP-1 degradative molecules [i.e., GLP-1(9-36), GLP-1(28-36)] may affect cardiovascular pathophysiology in diabetes. In this sense, DPP4 could also act as a direct mediator of endothelial dysfunction, via PAR2 activation and prostanoids release [39].

Interestingly, GLP-1 actions can be partially driven in an insulin-independent and myocardial specific fashion [12]. GLP-1R has been found in extra-pancreatic tissue like heart, and thus, GLP-1 could increase myocardial glucose uptake independently of its ability to enhance insulin secretion [40]. Also, sitagliptin stimulated myocardial ^18^FDG uptake in non-diabetic patients with dilated cardiomyopathy, without altering glucose homeostasis [30]. We now demonstrate that GLP-1 ameliorated HF-associated insulin resistance in cultured cardiomyocytes by up-regulation of both sarcolemmal and endosome Glut4 isoforms. GLP-1 also overexpressed Glut4 mRNA expression after 12 h HF-incubation. However, GLP-1 did not alleviate Glut4 levels after HG incubation, possibly because HG can also bind Glut4 and might induce a negative feed-back regulation by itself [41] or by its deviation products (i.e., hexosamines) [42]. Thus, we hypothesize that GLP-1 may directly promote both Glut4 translocation and mRNA expression after HF in the myocardium, as previously seen in insulin resistant muscle and liver, via PI3K/Akt and/or AMPK activation [43, 44]. In fact, sitagliptin-treated hearts exhibited a moderate increase of phospho-Akt^476^ and phospho-AMPKα^496^ in parallel to Glut4 translocation. Accordingly, the enrichment of sarcolemmal Glut4 induced by GLP-1 in HF-incubated cardiomyocytes, was attenuated by a specific PI3K inhibitor. This data confirms the participation of the axes Akt/PI3K and AMPKα, likely activated by GLP-1R stimulation [42, 44].

However, some GLP-1 activities may be also ascribed to GLP-1(9-36). This non-insulinotropic peptide is present in the circulation at higher levels than GLP-1 [45], though it shows 100-fold lower affinity for pancreatic GLP-1R [46, 47]. Remarkably, GLP-1(9-36) exhibited similar effects to GLP-1 as anti-fibrotic/apoptotic factor on cardiomyocytes [10], and reduced cardiac injury in ischemia/reperfusion models by GLP-1R dependent or independent mechanisms [12]. Also, GLP-1(9-36) induced vasodilatation through nitric oxide formation [12], and protected against oxidation in cardiac and vascular cells [48–50]. Here we show that GLP-1(9-36) improved cardiomyocyte Glut4 expression and sarcolemmal translocation after HF, in a similar way than GLP-1. GLP-1(9-36) may also activate GLP-1R and downstream Akt/PI3K and AMPKα mediators. Thus, the sequential modification of GLP-1 by DPP-4 from an insulinotropic to an insulinomimetic hormone [i.e., GLP-1(9-36)] could be also beneficial for cardiovascular protection [46]. These data also supports the insulin-independent actions of incretins and support the major outcomes of GLP-1R agonists over DPP-4 inhibitors [51]. In addition, GLP-1(9-36) may yield to a variety of N-terminal cleavage products, such as GLP-1(28-36), with demonstrated anti-oxidative proprieties [47].

### Limitation of the study

The evaluation of FA assimilation by PET at the myocardia could have added important information to our quantification of FA-transporters and enzymes. However, due to the extremely short half-life (~ 20 min) of a labelled FFA (i.e., [^11^C]-FFA) and the distance to the centre of synthesis, we could not achieve this approach. Also, despite we confirmed a weight-neutral effect of sitagliptin, it also reduced the food intake by 13% (p < 0.05), which may have contributed to the results.

## Conclusions

Since cardiac dysfunction is dramatically increasing in T2DM patients, new insulinomimetic-based interventions with cardioprotective proprieties are required. In this regard, sitagliptin improved hyperglycemia and insulin resistance in GK rats, and also, it enhanced cardiac glucose assimilation in detriment of excessive FA utilization and likely lipotoxicity. The sitagliptin-stabilized incretin, GLP-1, could inversely regulate sarcolemmal location of Glut4 and FAT/CD36 in cardiomyocytes via PI3K/Akt and AMPKα, but similar actions could be also triggered by GLP-1(9-36) metabolite. Therefore, these effects may occur independent of insulin regulation, opening the therapeutic utility to other cardiomyopathies different than DCM.
